# Real world outcomes of combination and timing of immunotherapy with radiotherapy for melanoma with brain metastases

**DOI:** 10.1002/cam4.3716

**Published:** 2021-01-22

**Authors:** Justin T. Moyers, Esther G. Chong, Jiahao Peng, Hsin Hsiang Clarence Tsai, Daniel Sufficool, David Shavlik, Gayathri Nagaraj

**Affiliations:** ^1^ Division of Hematology and Oncology Department of Internal Medicine Loma Linda University Loma Linda CA USA; ^2^ Department of Internal Medicine Loma Linda University Loma Linda CA USA; ^3^ School of Public Health Loma Linda University Loma Linda CA USA; ^4^ School of Medicine Loma Linda University Loma Linda CA USA; ^5^ Department of Radiation Medicine Loma Linda University Loma Linda CA USA

**Keywords:** brain metastasis, immunotherapy, melanoma, NCDB, stereotactic radiosurgery, whole brain radiotherapy

## Abstract

**Introduction:**

Immunotherapy (IT) and radiotherapy (RT) have improved overall survival in patients with melanoma with brain metastasis (MBM). We examined the real‐world survival impact of IT and RT combination and timing strategies.

**Materials and Methods:**

From the facility‐based National Cancer Database (NCDB) data set, 3008 cases of MBM were identified between 2011 and 2015. Six treatment cohorts were identified: stereotactic radiosurgery (SRS) + IT, SRS alone, whole brain radiotherapy (WBRT) + IT, WBRT alone, IT alone, and none. Concurrent therapy was defined as IT given within 28 days before or after RT; nonconcurrent defined as IT administered within 28–90 days of RT. The co‐primary outcomes were propensity score‐adjusted overall survival by treatment regimen and overall survival by RT and IT timing.

**Results:**

Median overall survival (mOS) was performed for each treatment category; SRS +IT 15.77 m; (95%CI 12.13–21.29), SRS alone (9.33 m; 95%CI: 8.0–11.3), IT alone (7.29 m; 95%CI: 5.35–12.91), WBRT +IT (4.89 m; 95%CI: 3.65–5.92), No RT or IT (3.29 m; 95%CI: 2.96–3.75), and WBRT alone (3.12 m; 95%CI 2.79–3.52). By propensity score matching, mOS for SRS +IT (15.5 m; 95%CI: 11.5–20.2) was greater than SRS alone (10.1 m; 95%CI: 8.4–11.8) (*p* = 0.010), and median survival for WBRT +IT (4.6 m; 95%CI: 3.4–5.6) was greater than WBRT alone (2.9 m; 95%CI: 2.5–3.5) (*p* < 0.001). In the SRS +IT group, 24‐month landmark survival was 47% (95%CI; 42–52) for concurrent versus 37% (95%CI; 30–44) for nonconcurrent (*p* = 0.40).

**Conclusion:**

Those who received IT in addition to WBRT and SRS experienced longer survival compared to RT modalities alone, while those receiving concurrent SRS and IT trended toward improved survival versus nonconcurrent therapy.

## Graphical abstract

In our study of the National Cancer Data Base (NCBD), addition of immunotherapy to radiation therapy improves survival in patients with melanoma with brain metastases when added to SBRT or WBRT, with the best survival observed for IT with SBRT. Concurrrent versus Noncurrent immunotherapy combined with radiation had no significantly different survival.

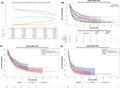

## 1 INTRODUCTION

The incidence of melanoma is increasing globally^1^ with a lifetime risk of 1 in 63 in the United States.^2^ Although it is the least common skin malignancy, it accounts for 73% of skin cancer‐related deaths.^3^ The incidence of brain metastasis in metastatic melanoma is as high as 50%.^4^, ^5^ The historic prognosis of melanoma with brain metastasis (MBM) has been poor at less than 5 months.^4^ Newer treatment strategies with single modality treatments have greatly improved outcomes through surgery, stereotactic radiosurgery,^6^ targeted therapy,^7^ and immunotherapy.^8^, ^9^

The clinical trajectory of metastatic melanoma was changed when immune checkpoint inhibitors (ICI) entered the immunotherapy landscape. Ipilimumab was the first ICI to be approved in 2011 following a phase 3 trial illustrating significant survival benefit.^10^ It was followed shortly thereafter by single agent nivolumab,^11^ pembrolizumab,^12^ and combination nivolumab and ipilimumab.^13^ Subsequently, attention has been given to examine the efficacy of ICI for patients with MBM in multiple phase II trials. Pembrolizumab achieved an objective intracranial response of 29.7% (*n* = 11/37) in asymptomatic brain metastases in PD‐L1 ≥ 1%.^14^, ^15^ Two sentinel studies examined the combination of nivolumab and ipilimumab in non‐irradiated asymptomatic brain metastasis. Long et al. compared nivolumab and ipilimumab to nivolumab; the intracranial benefit of 46% (*n* = 16/35) was noted for the combination and 20% (*n* = 5/25) in the single‐agent nivolumab group.^16^ Tawbi et al. found a rate of 57% intracranial benefit (*n* = 52/94) with an estimated 12‐month survival rate of 81.5% for the combination of nivolumab and ipilimumab while median overall survival data are yet to be published.^8^

While prospective trials on combinatorial strategies utilizing IT and localized RT are ongoing, single‐institution retrospective studies have shown significantly improved survival when IT is added to SRS with median overall survival ranging from 18.3 to 21.3 months.^17^, ^18^, ^19^ Our primary focus was to evaluate the survival benefit of various treatment strategies with IT and RT in MBM in this large database. Furthermore, the appropriate timing of IT relative to RT has remained a clinical conundrum. In an animal model study, mice treated with Ipilimumab and RT had significantly reduced tumor growth compared to single modality therapy.^20^ Using this knowledge combined with the half‐life of Ipilimumab of 14.7 days,^21^ Skrepnik et al. hypothesized the optimal timing of immunotherapy to be 6 days before to 14 days following radiation therapy.^22^ Two meta‐analyses have shown superior survival outcomes when radiation is given concurrently within 4 weeks before or after immunotherapy for MBM.^23^, ^24^ We, therefore, set out to determine the impact of timing of IT with RT on survival in patients with MBM as our co‐primary outcome in the NCDB cohort.

## 2 METHODS

This report follows the STROBE reporting guidelines for cohort studies.

### 2.1 Data source

The National Cancer Database (NCDB), established in 1989, is a nationwide, facility‐based, comprehensive clinical surveillance resource oncology data set that currently captures 52% of all melanoma cases and 72% of all newly diagnosed malignancies in the US annually.^25^ The NCDB is a joint project of the American Cancer Society and the Commission on Cancer of the American College of Surgeons. By Waiver of Determination, Loma Linda University Medical Center did not require IRB approval (IRB#5190177).

### 2.2 Study population

This retrospective cohort study used melanoma cases registered in the National Cancer Database (NCDB) between January 1, 2011 and December 31, 2015 as part of the Spring 2019 participant user file release and analyzed from November 1, 2019 to April 30, 2020.

We queried the database to identify patients with Stage IV melanoma and those with documented brain metastases. The co‐primary outcomes were survival by receipt of therapy modalities and the effect of timing of IT with RT. Patients were divided into six treatment cohorts: IT with stereotactic radiosurgery (SRS +IT), SRS alone, IT alone, WBRT with IT (WBRT +IT), WBRT alone, and none. Analysis of immunotherapy receipt was limited to IT given as first line therapy and radiation modality was limited to radiation volume given to the brain. SRS was defined as radiation modality of gamma knife, linear accelerator radiosurgery, protons, radiosurgery not otherwise specified (NOS), or undefined modality with documented fraction dose of 500 cGy or greater and 5 or less fractions. WBRT was defined as treatment modality of IMRT, conformal 3D‐radiotherapy, or undefined modality with fraction dose of less than 500 cGy or more than 5 fractions. We excluded those with insufficient documentation to determine receipt of immunotherapy or modality of radiation. Concurrent treatment with radiotherapy and immunotherapy was defined as first dose of IT administered 28 days before or after RT; non‐concurrent therapy was defined as IT given 29–90 days before or after RT.

### 2.3 Statistical analysis

Covariates examined between the immunotherapy groups included age, gender, year of diagnosis, Charlson‐Deyo‐comorbidity‐index (CDCI), primary payer, facility geographic region, facility location, facility type, education, income levels, and receipt of chemotherapy. Facility location were defined as metropolitan for facility in county with population greater than 250,000 versus suburban for facilities in county with population less than 250,000 versus rural defined for facilities in county with a population less than 2500. Education and Income levels were classified by the 5‐year estimates of the 2012–2016 update of the United States Census Bureau's American Community Survey Results for cases’ ZIP code. Education level is defined by the percentage quartile of high‐school graduates and median income by the upper or lower 50^th^ percentiles of income. The primary payer as registered at the time of diagnosis was used to determine insurance status.

Continuous variables are presented as the mean and standard deviation and compared using one‐way analysis of variance. Categorical variables are presented as frequency and percent with comparisons performed by Chi‐Squared analysis.

Univariable and multivariable time‐dependent cox‐proportional hazard models was used to estimate hazard ratios with 95% confidence intervals (C.I.) to identify significant predictors of mortality. Variables included in the model were age, sex, race, year of diagnosis, income percentile, insurance status, location, presence of extracranial disease, Charlson‐Deyo‐comorbidity‐index, receipt of chemotherapy, and the six treatment groups. In the time‐dependent cox model, the standard cox model was expanded by incorporating time‐dependent covariables with treatment groups dynamically updated during follow up depending on when the treatment began. Combined treatment was indicated only after the second treatment began. The Cox proportional hazards assumption was evaluated. Significant non‐proportionality of hazards was present and a single time interaction term was retained in the model for one covariate. The Kaplan–Meier (KM) method was used to estimate survival time of the treatment groups. The time variable for KM was calculated as the difference in days between start of intervention of RT or IO and death or last contact for single treatment. For combined treatment the time variable was the difference in days between the start of the second treatment and death or last contact. Survival times are presented with 95% confidence intervals and compared using log rank method and adjusted log‐rank method for propensity matching. Two‐sided *p *< 0.05 indicated statistical significance.

Propensity score matching (PSM) was utilized to estimate overall survival in MBM treated with RT modalities plus Immunotherapy versus RT modalities alone. PSM was performed based on unbalanced demographic factors and disease factors including age, sex, race, year of diagnosis, income quartile, facility location, facility type, insurance payer, Charlson‐Deyo score, and presence of extracranial metastasis. For PSM, one to up to four matching using the Greedy nearest neighbor algorithm was performed. A caliper width was narrowed in a stepwise fashion until the covariate distributions were balanced after matching. A caliper of 0.2 was finally used.

All statistical analyses were performed utilizing SPSS ® statistical software (version 25, IBM), SAS software (version 9.4; SAS Institute Inc.), and Microsoft Excel ® (Redmond, WA, USA).

## 3 RESULTS

### 3.1 Baseline characteristics of data sample

**FIGURE 1:**
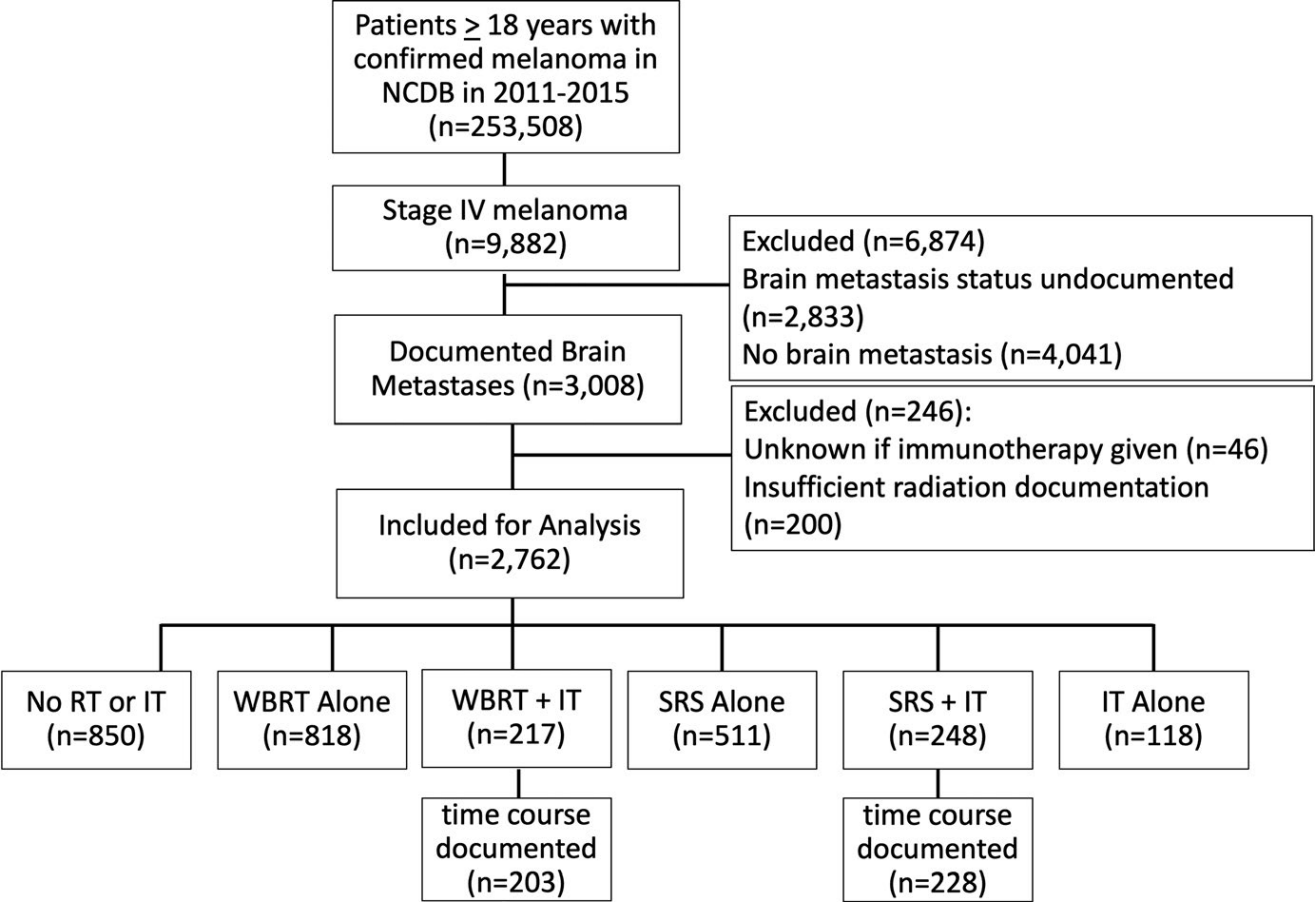
CONSORT diagram of case allocation

The CONSORT diagram detailing patient allotment is shown in Figure 1. Among 295, 200 melanoma diagnoses in the database between 2011 and 2015, 9882 stage IV diagnoses were identified with 3008 of those having documented brain metastases. The Incidence of documented brain metastases at presentation was 30.4% in this cohort.

**FIGURE 2:**
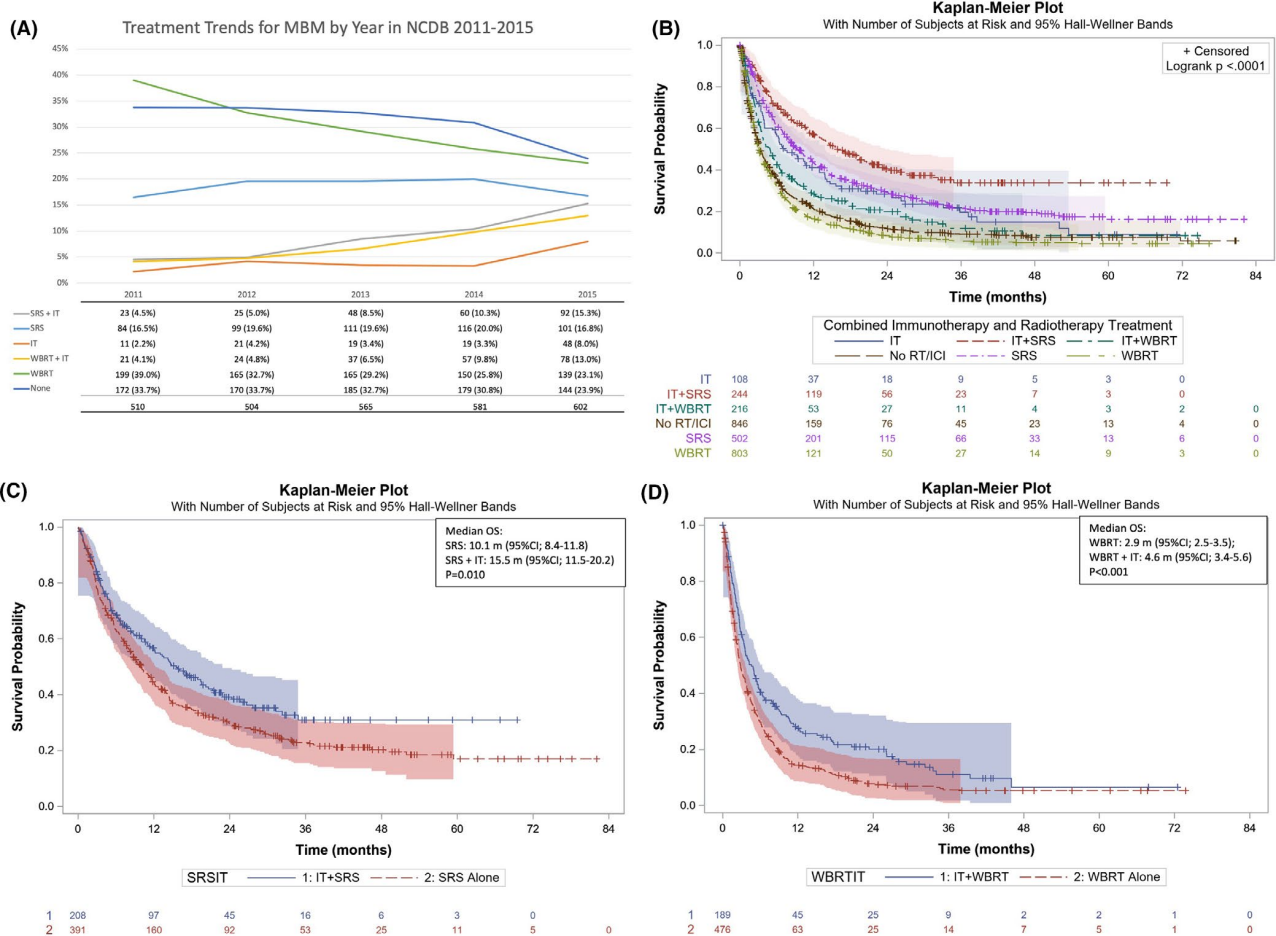
(A) Percentage of patients receiving each treatment by year. IT, Immunotherapy, SRS, Stereotactic radiosurgery, WBRT, whole brain radiotherapy. (B) Overall Survival by treatment regimen. IT, Immunotherapy, SRS, Stereotactic radiosurgery, WBRT, whole brain radiotherapy. (C) Propensity score matched overall survival by SRS +IT versus SRS. (D) Propensity score matched overall survival by WBRT +IT versus SRS

Table 1 lists the demographic data for the sample comparing cohorts of different treatment modalities. The mean age of the study population was 63.5 years. 97.5% of the population identified as white and 72% as male. Figure 2A, shows trends over study time frame with groups utilizing IT increasing over time while WBRT without IT decreasing in utilization.

**TABLE 1:** Demographic Data by Treatment Regimen

	Total N (%)	SRS +IT	SRS	IT	WBRT +IT	WBRT	No RT or IT	Statistical significance
	2762	248 (9)	511 (19)	118 (4)	217 (8)	818 (30)	850 (31)	
Age at Diagnosis								
Mean	63.5 (13.8)	60.7 (13.9)	62.8 (14.0)	61.8 (14.0)	60.6 (13.1)	62.7 (13.4)	66.5 (13.8)	*p* < 0.001
Gender								
Male	1981 (72)	175 (71)	364 (71)	86 (73)	168 (77)	594 (73)	594 (70)	*p* = 0.356
Female	781 (28)	73 (29)	147 29)	32 (27)	49 (23)	224 (27)	256 (30)	
Race								
White	2692 (97.5)	247 (99.5)	493 (97)	116 (98)	212 (98)	798 (98)	826 (97)	*p* = 0.201
Non‐White	70 (2.5)	1 (0.5)	18 (4)	2 (2)	5 (2)	20 (2)	24 (3)	
Charlson‐Deyo‐Comorbidity‐Index								
0	1962 (71)	205 (83)	378 (74)	99 (84)	165 (76)	548 (67)	567 (67)	*p* < 0.001
1	517 (19)	40 (16)	86 (17)	11 (9)	35 (16)	167 (20)	178 (21)	
2	177 (6)	3 (1)	31 (6)	6 (5)	11 (5)	64 (8)	62 (7)	
>=3	106 (4)	0 (0)	16 (3)	2 (2)	6 (3)	39 (5)	43 (5)	
Insurance Status								
Uninsured	148 (5)	6 (2)	20 (4)	4 (3)	10 (5)	50 (6)	58 (7)	*p* < 0.001
Private Payer	1040 (38)	130 (52)	233 (46)	51 (43)	97 (45)	292 (36)	237 (28)	
Medicare, Medicaid, Government	1527 (55)	111 (45)	249 (49)	61 (52)	105 (48)	467 (57)	534 (63)	
Undocumented	47 (2)	1 (0.4)	9 (2)	2 (2)	5 (2)	9 (1)	21 (3)	
% Without High School Degree								
=<10.8%	1559 (56)	169 (68)	309 (61)	71 (60)	128 (59)	457 (56)	425 (50)	*p* < 0.001
=>10.9%	1166 (42)	78 (32)	198 (39)	46 (39)	89 (41)	349 (43)	406 (48)	
Undocumented	37 (1)	1 (0.4)	4 (1)	1 (1)	0 (0)	12 (2)	19 (2)	
Median Income								
<=$50,353	1011 (37)	56 (23)	179 (35)	32 (27)	78 (36)	316 (39)	350 (41)	*p* < 0.001
>=$50,354	1709 (62)	190 (77)	327 (64)	85 (72)	138 (64)	488 (60)	481 (57)	
Undocumented	42 (2)	2 (1)	5 (1)	1 (1)	1 (1)	14 (2)	19 (2)	
Facility Type								
Community Based	1134 (41)	64 (26)	151 (30)	44 (37)	90 (42)	390 (48)	395 (47)	*p* < 0.001
Academic/Integrated Cancer Network	1478 (54)	160 (65)	329 (64)	66 (56)	113 (52)	387 (47)	423 (50)	
Undocumented	150 (5)	24 (10)	31 (6)	8 (7)	14 (7)	41 (5)	32 (4)	
Facility Location								
Metropolitan	2203 (80)	218 (88)	402 (79)	94 (80)	172 (79)	643 (79)	674 (79)	*p* = 0.208
Suburban	420 (15)	22 (9)	85 (17)	16 (14)	33 (15)	134 (16)	130 (15)	
Rural	69 (3)	3 (1)	9 (2)	2 (2)	7 (3)	22 (3)	26 (3)	
Undocumented	70 (3)	5 (2)	15 (3)	6 (5)	5 (2)	19 (3)	20 (2)	
Extracranial Metastases								
Yes	505 (59)	160 (65)	259 (51)	86 (73)	161 (74)	480 (59)	505 (59)	*p* < 0.001
No	328 (39)	85 (34)	243 (48)	31 (26)	55 (25)	320 (39)	328 (39)	
Unknown/Undocumented	17 (2)	3 (1)	9 (2)	1 (1)	1 (1)	18 (2)	17 (2)	
Radiation Characteristics								
Median Regional Dose (cGy)		2000 (810)	2100 (910)		3000 (660)	3000 (830)		
Median # Treatments		1 (1.4)	1 (2.6)		10 (3.3)	10 (4.5)		
Chemotherapy Given								
Yes	751 (31)	28 (4)	219 (29)	15 (2)	29 (4)	306 (41)	154 (22)	*p* < 0.001
No	1596 (66)	199 (87)	245 (52)	84 (82)	166 (83)	383(54)	519 (74)	
Unknown	69 (3)	5 (3)	17 (2)	3 (4)	8 (2)	69 (3)	33 (5)	

### 3.2 Survival and time to event analysis

Those without brain metastasis had median OS of 14.7 m (95%CI: 13.9–15.5 m) versus 6.0 m (5.6–6.3) for those with brain metastasis (*p* < 0.01).

When overall survival times were compared between the six categories of treatment as in Figure 2B, median survival was longest for those receiving both SRS and IT (15.77 m; 95%CI 12.13–21.29) with survival times decreasing in order of SRS alone (9.33 m; 95%CI: 8.0–11.3), IT alone (7.29 m; 95%CI: 5.35–12.91), WBRT +IT (4.89 m; 95%CI: 3.65–5.92), No RT or IT (3.29 m; 95%CI: 2.96–3.75), and WBRT alone (3.12 m; 95%CI 2.79–3.52). PSM was then utilized to observe the effect on survival when IT is added to RT. By PSM, median OS for SRS +IT (15.5 m; 95%CI: 11.5–20.2) was greater than survival of SRS alone (10.1 m; 95%CI: 8.4–11.8) (*p* = 0.010) Figure 2C. PSM showed improved median survival for WBRT +IT (4.6 m; 95%CI: 3.4–5.6) compared to WBRT alone (2.9 m; 95%CI: 2.5–3.5) (*p* < 0.001). Figure 2D.

### 3.3 Time‐delay adjusted cox‐regression model for overall survival

**FIGURE 3:**
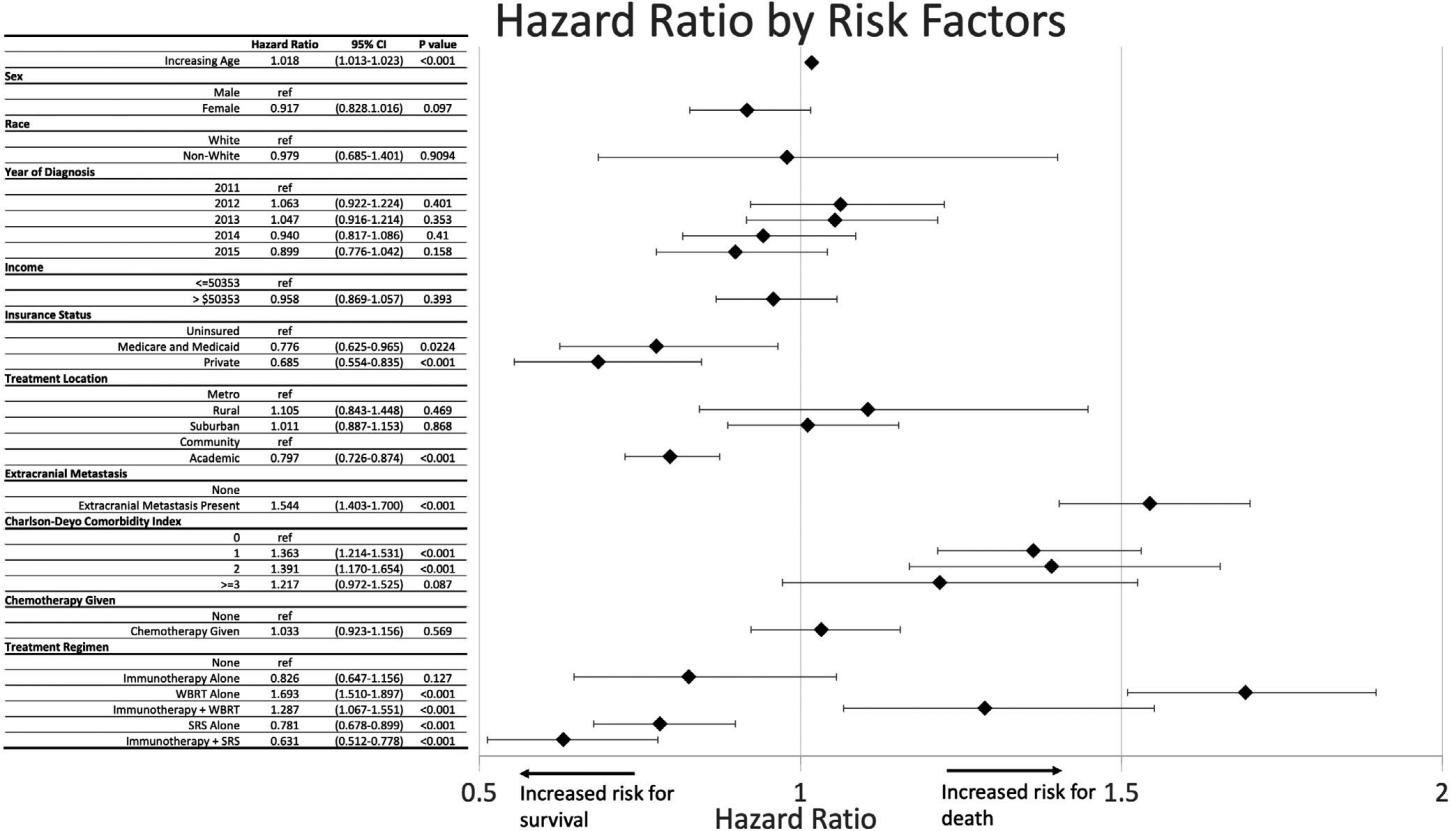
Cox Regression for survival. HR less than 1 show benefit for increased survival, whereas HR greater than 1 are increased hazard for death. HR, Hazard ratio; IT, Immunotherapy; SRS, Stereotactic radiosurgery; WBRT, whole brain radiotherapy

In the multivariable cox regression model, we did not find a statistically significant risk or benefit for survival based on sex, year of diagnosis, income percentile, ZIP‐code of residence, or use of chemotherapeutic agents. Increasing age (HR: 1.018; 95%CI 1.013–1.023), presence of extracranial metastasis (HR 1.544; 95%CI 1.403–1.700), and increased Charlson‐Deyo score of 1 (HR: 1.363; 95%CI 1.214–1.531) or score of 2 (HR 1.391; 95%CI: 1.170–1.654) were all associated with a hazard for death. Compared to uninsured patients, insurance with government based (HR: 0.776: 95%CI 0.625–0.965) and private payer (HR:0.685; 95%CI: 0.554–0.835) as well as treatment at an academic center compared to community center (HR:0.797; 95%CI:0.725–0.874) were all associated with a survival benefit. With regards to treatment modalities used, WBRT (HR 1.693: 95%CI: 1.510–1.897) and WBRT +IT (HR 1.287: 95%CI: 1.067–1.551) were associated with an increased risk of death. SRS (HR 0.781; 95%CI:0.678–0.899) and SRS +IT (HR 0.631; 95%CI:0.512–0.778) were associated with a survival benefit. Immunotherapy alone was associated with a non‐statistically significant survival benefit (HR:0.826; 95%CI:0.647–1.27). Results are summarized in the Forest Plot in Figure 3.

### 3.4 Treatment sequencing of combination modalities

**FIGURE 4:**
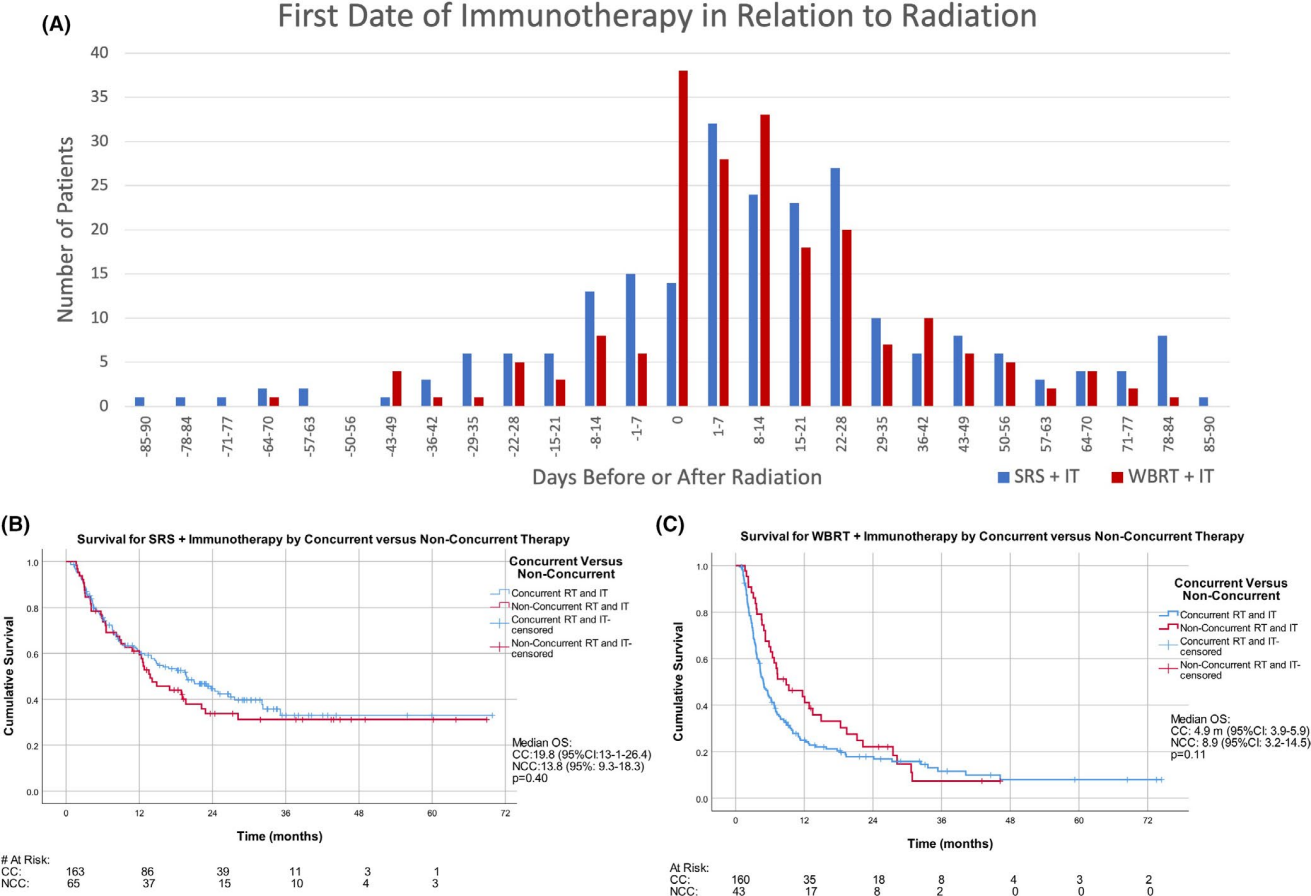
(A) First day of immunotherapy prior to first day of radiation (as negative values) or after the last day of radiation (as positive values). First day of immunotherapy given during course of radiation is listed as day 0. (B) Concurrent (CC) versus Non‐Concurrent (NCC) combination immunotherapy and radiation for SRS +IT. (C) Concurrent (CC) versus Non‐Concurrent (NCC) combination immunotherapy and radiation for WBRT + IT

In the combination IT and RT groups, 436 cases had documented time course between first dose of IT and RT to determine concurrent versus nonconcurrent therapies. IT was given concurrent with RT in 71.4% of SRS (*n* = 163/228) and 78.8% of WBRT (*n* = 160/203) treated patients. In SRS +IT cohort, survival trended longer for concurrent compared to nonconcurrent treatments (19.8 v 13.8 m; *p* = 0.35) with higher 24‐month landmark survival for concurrent therapy (47%; 95%CI: 42–52) versus nonconcurrent therapy (37%; 95%CI: 30–44) Figure 4B. However, these were not statistically significant. Survival was similar in WBRT+IT group for concurrent versus nonconcurrent groups (3.9 v 8.9 m; *p* = 0.11) with 24‐month landmark survival nearly equivalent 20% (95%CI 16–24) and 21% (95%CI 14–28) for the respective groups Figure 4C.

## 4 DISCUSSION

The NCDB cohort allows for large retrospective analysis of cases providing substantially improved generalizability compared to smaller single‐institution studies. Brain metastasis was documented at diagnosis of Stage IV melanoma in 30.4% of patients in the NCDB cohort. This differs from previously reported studies showing that 40–50% of patients develop brain metastasis during their disease course. This is likely due to the database not capturing the development of metastasis later in the course of the disease after time of diagnosis and instances of incomplete documentation in the database.^26^ Among all cases, regardless of treatment, the survival for those with melanoma with brain metastasis in NCDB between 2011 and 2015 was significantly decreased by nearly 9 months compared to those without brain metastasis (mOS 6.0 versus 14.7 months).

We found a longer survival for those selected to receive IT in addition to SRS with propensity score matched median OS was 15.5 versus 10.1 months in patients selected to receive SRS alone, which support the findings of previously reported smaller institution‐based retrospective studies.^27^, ^28^ Early reports of ipilimumab and radiation therapy found a median survival of 18.3–28.8 months compared to 4.9–6.8 months in patients who received radiation only.^17^ Patients who received SRS and Pembrolizumab were noted to have higher intracranial response rate (ICRR) of 70%, whereas patients who had undergone SRS and ipilimumab had a higher ICRR compared to SRS alone (ICRR 32% versus 22%).^29^ A prospective cohort study echoed the significance of dual‐modality therapy, and have shown that patients receiving radiation and immunotherapy have an OS of 13.2–16.8 months.^30^ It is hypothesized that radiation creates a synergistic effect with IT through the abscopal effect by inducing response against untreated micro‐metastasis and extracranial lesions.^29^, ^31^, ^32^, ^33^

We found patients who received WBRT alone had no beneficial effect with shorter overall survival compared to those in the no RT or IT group. These findings in melanoma echo the results of the landmark QUARTZ trial in Non‐Small Cell Lung Cancer wherein WBRT did not add survival benefit.^34^

While four immune checkpoint inhibitor regimens (Nivolumab, ipilimumab, nivolumab and ipilimumab, and pembrolizumab) were approved for use during our study's timeframe, surveys on community practice treatment trends favored ipilimumab use with progressive community use of other regimens.^35^ The survival for single‐modality IT for MBM was found to be 7.29 m (mOS 95%CI: 5.35–12.91) in our cohort which is comparable to the 7.0 months seen in the Phase II study of Ipilimumab, which is the likely regimen the majority of patients included received.^9^ However, upfront anti‐PD1 or combination anti‐PD1 with anti‐CTLA4 has since become the standard practice in patients with MBM and our cohort's observed survival times were less than the survival times achieved with these regimens: median OS of pembrolizumab at 17.6 months and 18.5 months for nivolumab with the combination nivolumab and ipilimumab achieving 75% overall survival at 18‐months.^16^, ^36^, ^37^

While our study was not powered to compare single‐modality SRS and IT, they had similar median OS. There are no clinical trials yet that have randomized treatment between IT and RT.

Multiple cohort studies comparing concurrent therapy to non‐concurrent therapy have shown a significantly improved local control and decrease in disease volume, but there was only a trend toward increased survival observed without reaching statistical significance.^22^, ^38^, ^39^ A retrospective study of 46 patients by Kiess et al showed a statistically significant improvement in survival for Ipilimumab given after SRS compared to Ipilimumab given before SRS, the median time between SRS and IT was 3 months suggesting most patients did not receive concurrent therapy by our definition. Furthermore, there was no improvement in recurrence free survival between groups calling into question the true survival benefit achieved.^40^ A multi‐institutional retrospective cohort study by the German Dermatologic Oncology Group (DeCOG) examined the effect of preceding IT with RT.^41^ This study analyzed a sub‐cohort of patients with brain metastasis (*n* = 233) and did not find those preceding either anti‐PD‐1 or anti‐CTLA‐4 therapy with radiation provided a progression free survival or overall survival difference.

A meta‐analysis by Lehrer et al. of 17 studies including 534 patients looked at treatment sequencing of 300 of those patients.^24^ With an identical definition of concurrent therapy, they found an increased survival at 12‐month landmark analysis (64.6% versus 51.6%, *p* < 0.01); whereas we found 12‐month survival for SRS +IT to be similar between concurrent (63%; 95% CI: 59–67) and non‐concurrent (63%; 95%CI: 57–69%) groups. While the number of patients in our study available for time sequencing analysis for SRS and IT was smaller (228 versus 300), only 28% (*n* = 63) of patients in the meta‐analysis were treated concurrently with SRS and IT while 72% (*n* = 163) of patients in our NCDB cohort underwent concurrent treatment.

The management of brain metastases has been examined in the prior NCDB studies; with increasing use of SRS to treat brain metastasis between the years 2000–2014 in several cancer types including melanoma.^42^ Previous studies of the NCDB by Stokes et al. of diagnosis years 2010–2013, Gabani et al. for cases diagnosed between 2011 and 2013, and Iorgulescu et al. on survival analysis of diagnosis years 2011–2014, each found survival benefits to immunotherapy when added to RT.^43^, ^44^, ^45^ These analyses of cases were early in the time of IT use found overall survival to be improved when IT was added to radiation, but only one compared the survival between combination strategies with radiation and none examined the overall survival of single‐modality IT or the timing of IT with radiation in their analysis.

Due to the favorable results seen with combined radiation and immunotherapy, multiple prospective clinical trials are currently being conducted to assess safety, efficacy, and appropriate timing of combination.^46^, ^47^, ^48^, ^49^

### 4.1 Limitations

The results of our study must be interpreted within the limitations of the NCDB cohort. The NCDB is a retrospective database that does not cover the entire population. Furthermore, only first‐line systemic therapy is recorded. Immunotherapy given as second line may still improve survival^50^ but receipt would not be recorded in the database. The specific agent or agents used as IT is not recorded and could include ICIs, interleukins, or other biologic modifying agents. Similarly, the specific agent used for receipt of chemotherapy includes cytotoxic chemotherapy and targeted inhibitors (such as BRAF/MEK inhibitors). However, chemotherapy receipt did not affect survival in our multivariate analysis. Sociodemographic factors, disease factors, and treatment location are likely to have an effect on treatment modality given. And incomplete recording of patient data may cause under ascertainment of radiation therapy, which could increase bias to the null hypothesis.

In addition, the burden of disease, symptom burden, and number of lesions in both the brain and extracranial space at the time of presentation is not available from the data which would affect the treatment plan recommended for a given patient scenario. Furthermore, some patients underwent metastectomy during their course of treatment but the site of metastectomy is not recorded in the database. Importantly, we are only able to assess survival from analysis of database; safety and local control rates and disease‐specific survival cannot be assessed.^51^

While we utilized PSM to adjust survival comparisons in attempts to account for confounding variables, unrecorded variables as aforementioned could not be included in this adjustment.^52^, ^53^ Due to limitations of the methodology, we were unable to compare all six groups by PSM.

The data used in the study are derived from a de‐identified NCDB file. The American College of Surgeons and the Commission on Cancer have not verified and are not responsible for the analytic or statistical methodology employed, or the conclusions drawn from these data by the investigator.

## 5 CONCLUSION

Utilizing the NCDB we found that use of combined strategy of immunotherapy and radiation in melanoma with brain metastases has increased since the FDA‐approval for ICIs. Longer survival was seen for those who received immunotherapy in addition to both WBRT and SRS. Immunotherapy alone prolonged survival compared to no therapy but was not statistically significant. Non‐statistically significant improvement in the 24‐month landmark survival was observed when immunotherapy was given concurrently with SRS.

## CONFLICT OF INTEREST

JTM received travel compensation from Astellas pharmaceuticals. ECG, JP, DS, DS, and GN report no conflicts of interest.

## Data Availability

The data that support the findings of this study are available from National Cancer Database. Restrictions apply to the availability of these data, which were used under license for this study.
